# Meningitis-associated pneumococcal serotype 8, ST 53, strain is hypervirulent in a rat model and has non-haemolytic pneumolysin which can be attenuated by liposomes

**DOI:** 10.3389/fcimb.2022.1106063

**Published:** 2023-01-06

**Authors:** Annelies Müller, Cebile Lekhuleni, Sabrina Hupp, Mignon du Plessis, Lalaina Holivololona, Eduard Babiychuk, Stephen L. Leib, Denis Grandgirard, Asparouh I. Iliev, Anne von Gottberg, Lucy J. Hathaway

**Affiliations:** ^1^ Institute for Infectious Diseases, Faculty of Medicine, University of Bern, Bern, Switzerland; ^2^ Graduate School for Cellular and Biomedical Sciences, Faculty of Medicine, University of Bern, Bern, Switzerland; ^3^ Centre for Respiratory Diseases and Meningitis, National Institute for Communicable Diseases, Division of the National Health Laboratory Service, Johannesburg, South Africa; ^4^ School of Pathology, Faculty of Health Sciences, University of the Witwatersrand, Johannesburg, South Africa; ^5^ Institute of Anatomy, University of Bern, Bern, Switzerland

**Keywords:** *Streptococcus pneumoniae*, meningitis, serotype 8, pneumolysin, liposomes, hyperviruent serotype 8

## Abstract

**Introduction:**

*Streptococcus pneumoniae* bacteria cause life-threatening invasive pneumococcal disease (IPD), including meningitis. Pneumococci are classified into serotypes, determined by differences in capsular polysaccharide and both serotype and pneumolysin toxin are associated with disease severity. Strains of serotype 8, ST 53, are increasing in prevalence in IPD in several countries.

**Methods:**

Here we tested the virulence of such an isolate in a rat model of meningitis in comparison with a serotype 15B and a serotype 14 isolate. All three were isolated from meningitis patients in South Africa in 2019, where serotype 8 is currently the most common serotype in IPD.

**Results and Discussion:**

Only the serotype 8 isolate was hypervirulent causing brain injury and a high mortality rate. It induced a greater inflammatory cytokine response than either the serotype 15B or 14 strain in the rat model and from primary mixed-glia cells isolated from mouse brains. It had the thickest capsule of the three strains and produced non-haemolytic pneumolysin. Pneumolysin-sequestering liposomes reduced the neuroinflammatory cytokine response *in vitro* indicating that liposomes have the potential to be an effective adjuvant therapy even for hypervirulent pneumococcal strains with non-haemolytic pneumolysin.

## 1 Introduction

*S. pneumoniae* is a pathogen of global importance which can cause life-threatening invasive pneumococcal disease (IPD) including meningitis. Its capsular polysaccharide (CPS) defines the serotype and is used for the manufacture of pneumococcal vaccines. Of the currently known 100 pneumococcal serotypes, only 13 are included in the most commonly used pneumococcal conjugate vaccine (PCV), PCV-13. There is strong evidence that the CPS of this opportunistic pathogen influences disease severity (Kadioglu et al., 2008). Previous studies have shown associations between serotype and disease outcome both in patients (von Gottberg et al., 2013; Grabenstein and Musey, 2014; Iroh Tam et al., 2017; Balsells et al., 2018; Croucher et al., 2018) and in *in vivo* models (Hathaway et al., 2016; Jacques et al., 2020). In epidemiological studies, elevated case-fatality ratio (CFR) has been shown for serotypes 3, 6B, 9N, 11A, 16F, 19F, and 19A in adults (Grabenstein and Musey, 2014) and 3, 6A and 19F in children (Song et al., 2013; Müller et al., 2022).

Capsule thickness, differences in growth and possible associations of serotypes with inflammatory profile could all be reasons for differences in serotype related disease severity (Hathaway et al., 2012; Muller et al., 2020; Müller et al., 2021). Additionally, pneumococci also express pneumolysin (Ply), a cholesterol-dependent cytolysin, which triggers the host inflammatory response (Cockeran et al., 2002; Malley et al., 2003; Jefferies et al., 2007; Matthias et al., 2008; McNeela et al., 2010; González-Juarbe et al., 2017; Subramanian et al., 2019; Hupp et al., 2022). Pneumolysin of different strains have various degrees of haemolytic activity, with some completely lacking pore-forming activity (Badgujar et al., 2020). Liposomes composed of cholesterol and sphingomyelin have been designed to sequester Ply and therefore attenuate pneumococcal virulence (Henry et al., 2014) but we are not aware of any studies on the effectiveness of such liposomes on non-haemolytic pneumolysin. The mechanisms whereby specific serotypes and strains with different pneumolysin types could cause more severe disease than others are still largely unknown. Also, most studies aiming to understand serotype and non-serotype specific pneumococcal virulence mechanisms are either purely *in vitro, in vivo* or large epidemiological studies based on patient data and statistical models. Studies which compare current important clinical strains with available patient data directly in laboratory models within one study are scarce. Strains used in laboratory experiments are often extensively studied and characterized, such as strain D39, of serotype 2. However, in South African IPD patients for example, serotype 2 pneumococci are rare and numbers are decreasing (Müller et al., 2022), as observed worldwide (https://www.pneumogen.net/gps/index.html). In contrast, the prevalence of serotype 8, a non-PCV13 serotype, and specifically serotype 8 of multilocus sequence type (MLST) 53, is currently increasing in IPD cases both in Denmark (Hansen et al., 2021) and in Madrid, Spain (Sanz et al., 2020). Serotype 8 is also the most common serotype in the vaccine era in South African IPD patients (Müller et al., 2022) and in Switzerland (Oyewole et al., 2021). Therefore, in addition to laboratory strains, current clinically relevant serotypes should be investigated in *in vitro* and *in vivo* experiments, especially when considering serotype-specific differences in disease severity.

Here we studied currently circulating clinical pneumococcal strains and our aim was to compare *in vivo* data from an animal model infected with different pneumococcal serotypes of clinical importance. We chose three clinical isolates of different serotypes, isolated from South African meningitis patients. Serotype 8 was chosen, because it is the most common serotype in the vaccine era (Müller et al., 2022), serotype 15B because we previously found that it was associated with increased mortality, while serotype 14 was less frequently associated with mortality than the two other serotypes in our previous study (Müller et al., 2021). For these strains, clinical data and the patient CSF inflammatory profile was available. We looked at mortality, and CSF cytokine concentrations (IL-6, IL-8, TNFα, IL-10, IL-1β, IFN-γ) in three patients and in a pneumococcal meningitis model. We wanted to identify possible differences between the isolates in regards to disease severity. We analyzed capsule size *in vivo*, pneumolysin expression and haemolytic activity. We assessed disease severity by looking at mortality, hippocampal apoptosis, cortical damage and concentration of neurofilament light chain in CSF in the *in vivo* model. Understanding the mechanisms of pneumococcal virulence in humans and determining whether and how *in vivo* models represent the clinical situation could help in understanding mechanisms of pathogenicity, in turn helping the development of new treatment and vaccine strategies.

## 2 Methods and methods

### Bacterial strains

We used three isolates representing serotypes 8, 15B and 14 recovered from cerebrospinal fluid (CSF) of meningitis patients in 2019. The isolates were collected as part of the South African GERMS national laboratory-based surveillance program and kindly provided by the National Institute for Communicable Diseases (NICD) in Johannesburg. Serotype for all strains was confirmed by the Quellung reaction method (Statens Serum Institute, Copenhagen, Denmark) and whole genome sequencing. Additionally, the CSF of the patients infected with these isolates was collected as part of a larger study analyzing the severity of disease caused by different pneumococcal serotypes in the vaccine era (Müller et al., 2021). Therefore, basic patient data such as in-hospital outcome, status of human immunodeficiency virus (HIV) infection, age, and gender as well as the concentrations of the cytokines IL-6, IL-8, TNFα, IFN-γ, IL-10, IL-1β in patient CSF were known. A serotype 8 strain (58941) was chosen because it was found to be the most common serotype in IPD patients in South Africa in the vaccine era (Müller et al., 2022). The other two serotypes were chosen due to available CSF data and their association with mortality compared to serotype 8 (15B, strain 58331, significantly associated with higher mortality and serotype 14, strain 55927, associated with lower mortality) (Müller et al., 2021).

### Sample collection, storage and analysis of human CSF samples

CSF patient samples were collected at the request of the attending clinician. After completion of diagnostic procedures and after the presence of *S. pneumoniae* was confirmed by the local laboratory, the residual CSF was collected by the site surveillance officers. Surveillance officers were given a standard operating procedure (SOP) for the collection of CSF samples. They were requested to store the residual CSF samples on site until further transport (stored at -20°C or -80°C depending on availability of equipment). The residual CSF samples were then transported from the sentinel laboratory site of collection to the NICD. To avoid unnecessary freeze-thaw cycles all samples were transported on dry ice and immediately stored at -80°C upon arrival at the NICD until further multiplex analysis. Cytokines in human CSF were measured using a 39-plex microsphere based multiplex assays (Human Custom ProcartaPlex 39-plex cat. number PPX-39-MX9HJM4 Thermo Fisher Scientific, Waltham, MA, USA) including IL-6, IL-8, TNFα, IFN-γ, IL-10 and IL-1β as we have described previously (Müller et al., 2021). The assay was run according to the manufacturer’s instructions. Samples of 25 µl were measured undiluted and both the standards and CSF samples run in duplicate. Beads were acquired on a Bio-PlexTM 200 instrument (Bio-Rad) and the fluorescence signal for 50 beads per analyte measured. The data were analyzed with Bio-Plex manager (version 6.1, Bio-Rad) capable of generating a five-parameter logistic (5-PL) curve fit.

### Bacterial preparation for *in vivo* experiments

Bacteria were stored at – 80°C in Protect bacterial preservers (Technical Service Consultants, Heywood, U.K). For experiments, frozen stocks were prepared. Bacteria were plated on CSBA and grown overnight in a 37°C, 5% CO_2_ atmosphere from which three to 10 colonies were picked to inoculate 10 ml brain-heart infusion broth (BHI; Becton Dickinson and Company, le Pont de Claix, France). The culture was incubated until OD_600nm_ corresponded to mid-log phase. To ensure that all bacteria were in exponential growth phase, 1 ml of this culture was used to inoculate 9 ml fresh BHI and then grown again until OD_600nm_ corresponded to mid-log phase. The culture was then aliquoted (1 ml) and frozen at – 80°C in Eppendorf tubes. For inoculation, 1 ml of the frozen bacterial stock was inoculated into 9 ml fresh BHI and grown to mid-log phase. 0.5 ml of the culture was then inoculated into 9.5 ml fresh BHI and again grown to mid-log phase which was an OD_600nm_ of 0.3 for strain 58941 and an OD_600nm_ of 0.5 for strains 58331 and 55927. The resulting culture was centrifuged (3000 g, 4°C, 5 minutes) and washed with 10 ml 0.85% sodium chloride solution (NaCl). Following two wash steps, the bacteria were diluted to the OD_600nm_ corresponding to a bacterial concentration of 1 - 5 x 10^6^ colony forming units (CFU)/ml. The actual inoculation concentration was confirmed by performing serial dilution of the inoculation suspension, plating on CSBA, incubation overnight in a 37°C, 5% CO_2_ atmosphere and counting colonies the following day.

### Pneumococcal meningitis model

We used a well-established infant rat model of bacterial meningitis with modifications as described as follows (Leib et al., 2000). Bacteria were not passaged in animals prior to their use *in vivo*. A total of 36 11-day old male and female nursing Wistar rats along with their dams were obtained from Charles River (Sulzfeld, Germany). The dams were provided with tap water and pellet diet *ad libitum*. All animals were kept in a room at controlled temperature (22 ± 2°C) and natural light. The pups (average weight 25.0 ± 3.7 g) were infected intracisternally *via* a 32- gauge needle with an inoculum of 10 µl 0.85% NaCl containing 1 – 5 x 10^6^ CFU/ml live *S. pneumoniae*, resulting in a final inoculation of 10^4^ CFU. Bacterial strains 58941 (serotype 8, *n = 12 pups*), 58331 (serotype 15B, *n = 12*) and 55927 (serotype 14, *n = 12*) were randomly assigned to groups of 4 animals per experiment. The 3 experiments differed in the timing at which CSF was sampled and the first dose of antibiotics administered. Successful infection was tested by quantitative analysis of bacterial cultures in samples of CSF at either 17, 19 or 21 hours post infection (hpi). The bacterial concentrations were measured by performing serial dilutions of 5 µl of CSF in 0.85% NaCl which was plated onto CSBA plates, incubated overnight at 37°C with 5% CO2 and counted the next day. All animals received antibiotic therapy with ceftriaxone (Rocephine^®^, Roche Pharma, Basel, Switzerland; 100 mg kg^-1^ bid, i.p.) after CSF samples were collected for analysis at either 17, 19 or 21 hours and 8 hours after first dose. Severity of disease was assessed by weighing and clinical examination with a scoring at 0, 17 or 19 or 21 and 45 hpi depending on experiment. The scoring was performed as previously described (Leib et al., 2000) with the following scale: 1 = coma; 2 = does not stand upright after being turned on the back; 3 = stands upright within 30 s; 4 = minimal ambulatory activity, stands upright in <5 s; 5 = normal. For ethical reasons, animals with a score of 2 or less were immediately sacrificed by an overdose of pentobarbital (Esconarkon, Streuli, Uznach, Switzerland; 150 mg kg^-1^, i.p.). For CSF analysis, 10 to 30 µl of CSF was obtained by puncture of the cisterna magna. 5 µl was used for the quantitative analysis of bacterial cultures as described above, the rest was centrifuged (16 000 g, 4°C, 10 minutes) and the supernatant immediately frozen at – 80°C for later analysis of cytokines and neurofilaments, of which the cytokine analysis was prioritized. The pellet was used for analysis of capsule thickness. At 45 hpi, animals were sacrificed with an overdose of pentobarbital (150 mg kg^-1^ intraperitoneal), perfused with 4% paraformaldehyde (PFA) in PBS and the brains removed and fixed in PFA for histopathological evaluation.

### Histopathological evaluation of brain damage

We quantified brain damage in animals sacrificed at 45 hpi as previously described (Leib et al., 2000; Liechti et al., 2014). Neuronal apoptosis in the dentate gyrus of the hippocampus was assessed by counting cells with features of apoptosis (condensed, fragmented dark nuclei, apoptotic bodies). Apoptotic cells were counted in four slices spanning the hippocampus of both hemispheres. A mean value per animal was calculated from counting cells in three fields of view in each of the two blades of the dentate gyrus. A minimum of 16 brain sections per animal was used to assess cortical damage. Areas of decreased neuronal density or cortical necrosis were measured and are expressed as a percentage of total cortical volume investigated.

### Cytokine measurements in rats

For *in vivo* experiments with isolates from South African meningitis patients, we measured the concentrations of IL-6, TNFα, IFN-γ, IL-10, IL-1β and GRO KC CINC-1 [an IL-8-like cytokine in rats (Himi et al., 1997)] using a magnetic multiplex bead-based assay (Milliplex MAP Rat Cytokine/Chemokine Magnetic bead 6-plex Panel, cat. number RECYTMAG-65K-06, Merck-Millipore). The assays were run according to the manufacturer’s instructions. For each sample, a minimum of 50 beads was measured. 5 µl of collected rat CSF at either 17, 19, or 21 hpi depending on experiment and at 45 hpi for all experiments was diluted 1:5 for analysis. A value corresponding to the detection limit provided by the manufacturer and multiplied by the dilution factor was used for samples for which the value was below detection limit (IL-6 30.7 pg/ml; TNFα 1.9 pg/ml; IFN-γ 6.2 pg/ml, IL-10 2.7 pg/ml, IL-1β 2.8 pg/ml; and GRO KC CINC-1 19.7 pg/ml).

### Neurofilaments

The concentration of Neurofilament light chain (NfL) was determined in the CSF using an ELISA-based microfluidic system (ELLA, ProteinSimple, San Jose, CA, USA) with dedicated cartridges according to the manufacturer instructions, using 2 µl of CSF diluted to 50 µl with the calibrator buffer provided in the kit.

### Capsule size

The capsule size of pneumococcal isolates was measured by the FITC-dextran exclusion assay as described by Gates et al. (2004) with the modifications mentioned below. The method measures the zone of exclusion which correlates with capsule size. The pellet remaining after centrifugation of CSF samples as described above, was re-suspended in 2 µl FITC-dextran (2000 kDa, Sigma; 10 mg/ml in water), pipetted onto a glass slide and covered with a cover slip. The slide was viewed with a Zeiss Axio Imager M1 fluorescence microscope with a 100 x objective and the Zeiss AxioCam HRc camera was used for photographing slides. Capsule size was measured before infection of animals and at 17, 19 or 21 hpi depending on the experiment. Due to limited sample we viewed one slide per animal per time-point, adding up to a maximum of 4 images per serotype per experiment if bacteria were found on each slide. Capsule size was not measured after antibiotic treatment as no bacteria were found.

### Haemolysis assay

Bacteria were stored and prepared as described above. A sub-culture of 1 ml overnight culture and 9 ml fresh BHI was grown until OD_600nm_ corresponded to mid-log phase to ensure that all bacteria were in exponential growth phase. The bacteria were then centrifuged (4000 rpm, 10 min, 4°C). The supernatant was transferred and stored on ice until performance of assay. The pellet was resuspended in 2 ml phosphate buffered saline (PBS, pH = 7.0) and sonicated (Branson, SONIFIER 250; level 2.5, 6 X 10 seconds on ice) to release pneumolysin. Bacterial supernatants and sonicates were serially diluted and mixed 1:1 in round-bottomed 96-well plates with a 2% suspension of human erythrocytes (Interregionale Blutspende SRK AG Bern, Switzerland) in PBS (final reaction volume = 200 µl). The mixture was incubated for 30 minutes at 37°C and centrifuged 5 minutes at 4000 rpm. The supernatant was transferred to a new flat-bottomed 96-well plate and absorbance measured at OD_450nm_ (VICTOR Nivo^®^ Multimode Plate Reader, PerkinElmer). Controls consisted of a 0% haemolysis condition (PBS only) and a 100% haemolysis condition (dH_2_O only). Percent lysis was determined by normalizing absorbance values to the dH_2_O positive control (100% lysis) adjusted to the 0% lysis PBS negative control. Negative values of samples were considered to be 0% and values above 100% were considered to be 100% and changed accordingly for graphical representation.

### Preparation of primary mixed glial cultures

Primary mouse astrocytes and microglia were prepared from the cortices of newborn C57BL/6 mice (postnatal day (PD) 3-4) as mixed cultures. Briefly, the cortices of the newborn mice were dissociated into cell suspensions and transferred to cell culture flasks (75 cm^2^) (Sarstedt AG, Nuembrecht, Germany) coated with poly-L-ornithine (PLO, Sigma-Aldrich). The growth medium, DMEM (Dulbecco’s Modified Eagle’s Medium) (Gibco), was supplemented with 10% FCS (PAN Biotech, Aidenbach, Germany) and 1% penicillin/streptomycin (Gibco). Eleven to fourteen days after preparation, the cells were fully differentiated and ready to be used in the experimental procedures.

### Cytokine measurements in mouse primary mixed glial cultures

Murine mixed glia were plated on poly-L-ornithine-coated 24-well plates at a density of around 200,000 cells/well and subcultured for exactly one week after seeding. These populations consisted of astrocytes and microglia at approximately equal concentrations, as determined by immune staining of the mixed cultures (data not shown). Before treatments, the cells were washed once with PBS. TNF-α, IL-6 and CXCL2 (MIP-2, IL-8) were selected to determine the early pro-inflammatory response of the mixed glia to bacterial infection. The amount of cytokine release was measured after 24 h of continuous incubation of the cells with bacterial lysates (prepared as above) at 37°C and 5% CO_2_ in DMEM (80 mg/dl glucose, GlutaMax supplement, all from Gibco) without serum. We used a continuous challenge of the cells with lysates, to mimic the situation during infection. After incubation, the supernatants were centrifuged at 6000 g for 5 minutes to remove the residual cellular and bacterial debris. The samples were stored at -20°C until cytokines were measured with conventional sandwich enzyme-linked immunosorbent assay against natural mouse TNF-α, IL-6 (BioLegend ELISA MAX™, San Diego, CA, USA) and CXCL2/MIP-2 (R&D Systems, Minneapolis, MN, USA). The assays were carried out according to the manufacturer’s instructions. The absorbance was detected at 450_nm_ with a standard absorbance microplate reader (EL800, BioTek Instruments, Winooski, VT, USA).

### Pneumolysin ELISA

For determination of pneumolysin protein concentrations in the total lysates of different pneumococcal strains (equivalent to 5x 10^6^ CFU/ml), we used an established protocol based on a conventional double-antibody sandwich ELISA (Jacques et al., 2020). In order to induce lysis, fresh preparations of bacteria were incubated over night at room temperature with 100 U/ml of the bacteriolytic antibiotic penicillin (Gibco), with subsequent performance of bacterial CFU counts to ensure complete lysis. For the assay, high binding ELISA plates (Sarstedt) were coated with 1 µg per well mouse monoclonal PLY-4 antibody (abcam 71810) in 1x coating buffer (BioLegend) and incubated over night at 4°C. After washing, unspecific binding was blocked with 1% BSA/PBS for one hour at room temperature. The standard curve was established by 2-fold concentrations of purified recombinant pneumolysin (kindly provided by Timothy J. Mitchell) ranging from 3.125 ng/ml to 400 ng/ml. Standards and test samples were incubated for 2 hours at room temperature. 1 µg/ml rabbit polyclonal anti-PLY antibody (abcam 71811) was used as detection antibody (2 hours incubation) and goat-anti rabbit IgG alkaline phosphatase (abcam 97048) was added in a dilution of 1:5000 for 30 minutes as enzyme carrying antibody. Alkaline Phosphatase yellow pNNP was used as substrate and absorbance was measured at 450_nm_ (after 30 minutes incubation in the dark) with a conventional absorbance microplate reader (EL800, BioTek Instruments, Winooski, VT, USA). Recorded values of recombinant PLY standard were used to plot a curve of OD_450_ *vs* ng/ml and the concentrations of the test samples were calculated using the equation of the curve.

### Whole-genome sequencing

The clinical isolates of serotypes 8, 15B and 14 were sequenced and assembled. DNA was extracted from overnight brain heart infusion broth cultures using the QIAamp DNA mini kit (Qiagen, Inc., USA). The DNA extracts were quantified using the Qubit dsDNA BR assay kit (Life Technologies, Carlsbad, CA, USA). Multi-plexed paired end libraries were prepared using the Illumina Nextera DNA Flex Library Preparation kit (Illumina, San Diego, CA, USA). Whole-genome sequencing was carried out on an Illumina MiSeq platform.

### Genomic analysis and molecular typing

The Illumina paired-end reads were subjected to FastQC (https://github.com/s-andrews/FastQC) and trim_galore (https://www.bioinformatics.babraham.ac.uk/projects/trim_galore/) for quality check and read filtering, respectively. SPAdes (http://cab.spbu.ru/software/spades/) was used to generate *de novo* assemblies (Bankevich et al., 2012). The Pathogenwatch pipeline (https://cgps.gitbook.io/pathogenwatch/) was used to determine *in silico* serotypes using SeroBA (Epping et al., 2018); sequence types (ST) were identified from *de novo* assemblies (Page et al., 2016) using the PubMLST allelic scheme database.

### Characterization of serotype 8 strain pneumolysin

The assembled contigs of serotypes 8, 15B and 14 isolates were annotated using RAST (Aziz et al., 2008). The BLAST algorithm was used to identify the ply allele type. The non-haemolytic S. pneumoniae strain 01-1199 ply-5 reference sequence (https://www.ncbi.nlm.gov/search/all/?term=EF413926) was used in an amino acid alignment with strains 58941, 55927 and 58331 ply alleles for the identification of amino acid differences between haemolytic and non-haemolytic *ply* alleles.

### Statistics

All statistical analysis and creation of figures was performed using GraphPad Prism 8.4.3. To compare values of data we first performed a normality test. As the data were not normally distributed we used the Kruskal-Wallis test to test for differences and Dunn’s multiple comparison to test for significant differences between groups. Mortality between groups was compared by Gehan-Breslow-Wilcoxon test. A p-value of ≤ 0.05 was considered significant.

## 3 Results

### The serotype 8 isolate caused more mortality than the serotype 15B and 14 isolates in infant rats

**Figure 1:**
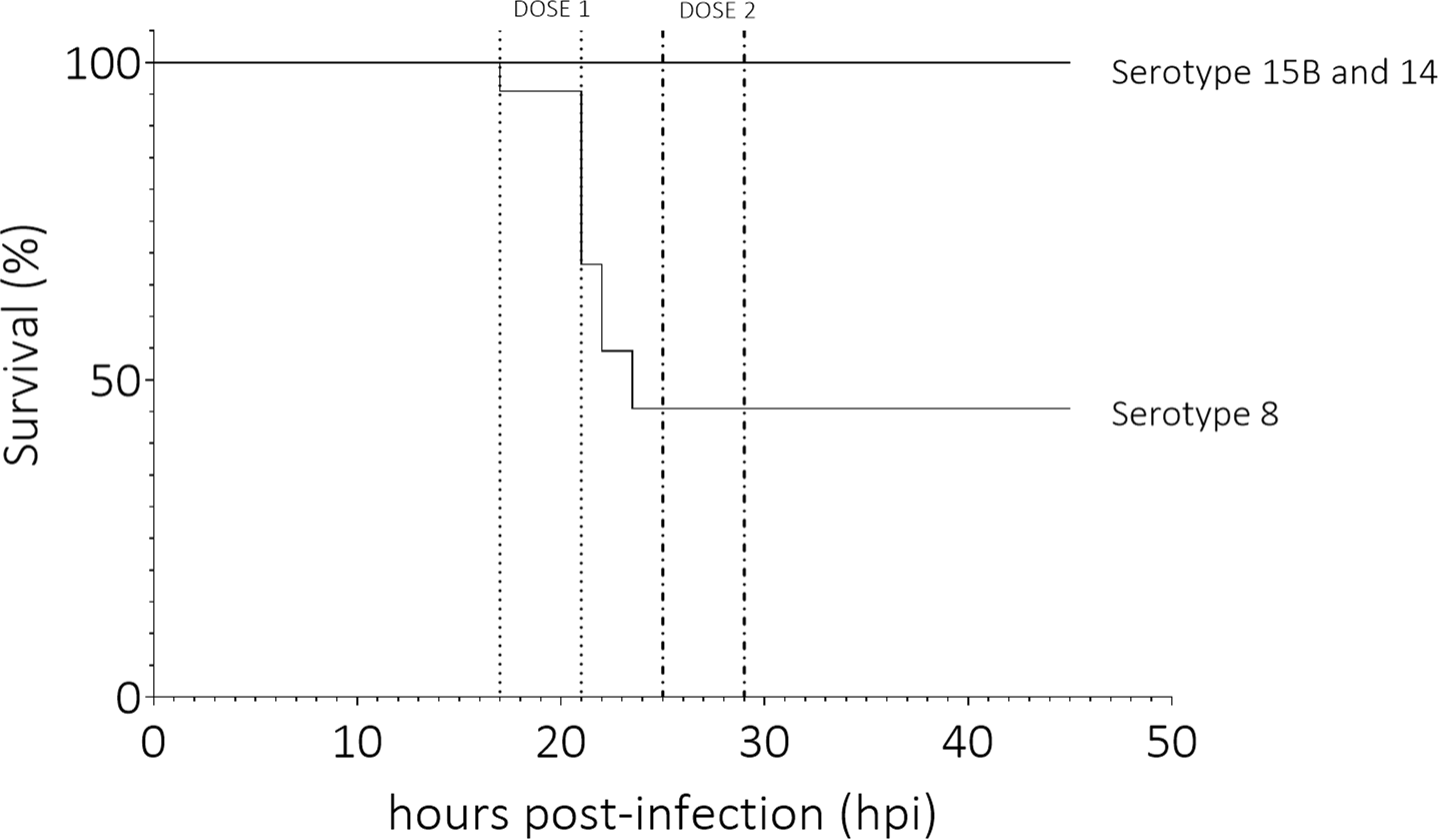
Survival (%) of animals infected with clinical pneumococcal strains of serotype 8, 15B or 14. Infant rats infected with the serotype 8 strain had significantly higher mortality than those infected with serotypes 15B or 14 (p-value < 0.0001). Dose 1 and 2 indicate the time frames in which the first and second dose of antibiotics were administered. Data represent 12 rats per serotype, total of 36 rats.

**Figure 2:**
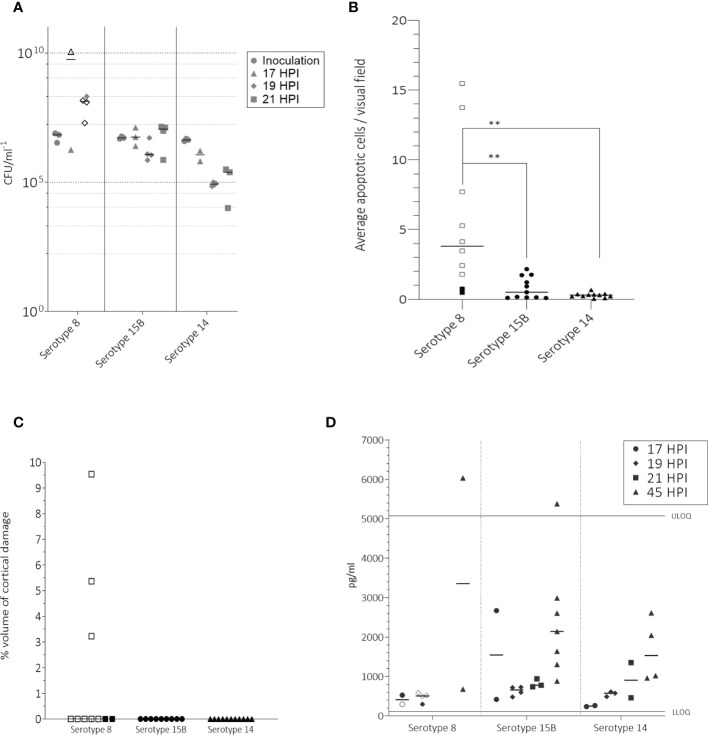
In the infant rat model following intracisternal administration of 1 – 5 x 10^6^ CFU/ml *S. pneumoniae* of serotypes 8, 15B or 14, bacterial titers at inoculation, 17, 19 and 21 hpi in the CSF **(A)**, number of apoptotic cells counted in the hippocampus **(B)**, volume of cortical injury **(C)** and neurofilaments (NfL) in the CSF at 45 hours post infection (hpi) **(D)**. **(A)** For inoculum, one point represents the average CFU/ml at the beginning of one experiment. At 45 hpi, after treatment with ceftriaxone no bacteria were detected. Animals infected with serotype 8 had significantly higher bacterial titres at 19 hpi than those infected with serotype 14 (p-value = 0.0051) **(B)** Animals infected with serotype 8 had significantly more (p-value < 0.05) apoptotic cells than those infected with either serotype 15B or 14. **(C)** Only animals infected with serotype 8 had detectable cortical injury. **(D)** Infected animals had a higher NfL concentration at 45 hpi than at 17, 19 or 21 hpi. Upper limit of quantification (ULOQ) and lower limit of quantification (LLOQ) of assay are indicated. Animals for which infection was unsuccessful are not shown. One point represents one animal. Open symbols represent animals which did not survive until the end of the experiment. ** = p-value ≤ 0.001.

We compared the effect of the three clinical isolates in an infant rat model of meningitis. Mortality rate of rats infected intracisternally with the serotype 8 pneumococcal strain was significantly higher than for those infected with 15B or 14 over 45 hours (p-value < 0.0001) (**Figure 1**). Infection was determined as successful if bacteria were recovered from the CSF at the time point of the first CSF tapping. For two animals which received the serotype 8 isolate, one which received the serotype 15B isolate and one which received the serotype 14 isolate infection was not successful (all tapping performed at 17 hpi). Mean bacterial titre for animals infected with serotype 8 was 5.6 x 10^9^ CFU ml^-1^ at 17 hpi and 1.2 x 10^8^ CFU ml^-1^ at 19 hpi (**Figure 2A**). CFU ml^-1^ determination at 21 hpi for animals infected with serotype 8 was not possible as we were not able to recover sufficient CSF from the animals. 3 of 4 animals in this group also died spontaneously and the fourth was euthanized due to a clinical score of 2. After treatment of animals with ceftriaxone, no bacteria were detected at 45 hpi in any of the animals. Animals infected with serotype 15B had mean bacterial titers of 1.2 x 10^6^ CFU ml^-1^ at 17 hpi, 2.0 x 10^6^ CFU ml^-1^ at 19 hpi, and 9.3 x 10^6^ CFU ml^-1^ at 21 hpi (**Figure 2A**). Serotype 14 infected animals had mean bacterial titres of 7.0 x 10^6^ CFU ml^-1^ at 17 hpi, 8.5 x 10^4^ CFU ml^-1^ at 19 hpi, and 2.0 x 10^5^ CFU ml^-1^ at 21 hpi (**Figure 2A**). At 19 hpi, animals infected with serotype 8 had a significantly higher bacterial titre than animals infected with serotype 14 (p-value = 0.0051). The average weight did not differ between the groups at inoculation (serotype 8: 25 g, sd ± 3.9; serotype 15B: 24 g, sd ± 3.3; serotype 14: 26 g, sd ± 4) or at 45 hpi for survivors (serotype 8: 28 g, sd ± 6.4; serotype 15B: 26 g, sd ± 3.3; serotype 14: 29 g, sd ± 4).

### The serotype 8 isolate caused more hippocampal apoptosis and cortical damage than the isolates of serotypes 15B and 14

To compare damage in the brain caused by the three clinical isolates, we determined the number of apoptotic cells in the hippocampus of all animals for which infection was successful. Serotype 8 caused significantly more apoptosis in the hippocampus than serotypes 15B (5.5 *vs* 0.8; p = 0.003) and 14 (5.5 *vs* 0.3; p = 0.001) (**Figure 2B**). More apoptosis was observed in the rats infected with serotype 15B than 14 but the difference was not significant (0.8 *vs* 0.3, p = 0.9) (**Figure 2B**). Cortical damage was not observed in any of the animals which survived until the end of the experiment (45 hpi) but was observed in 3 of the 8 animals infected with serotype 8 which did not survive until 45 hpi (**Figure 2C**).

### Pneumococcal serotypes 15B and 14 caused an increase of Neurofilament light chain in the CSF of infected rats

As a further indication of neuronal damage, we measured the presence of Neurofilament light chain (NfL) in the CSF of rats at the time point of first CSF tapping and at 45 hpi. We observed that the concentration of NfL in the CSF was slightly higher when tapping was performed at 19 or 21 hpi than when performed at 17 hpi, and higher at 21 hpi compared to 19 hpi, regardless of the infecting serotype. We also observed that after ceftriaxone had been administered, the concentration of NfL at 45 hpi was higher than at the time points before administration of ceftriaxone (**Figure 2D**). The differences were not significant due to the small amount of data. We were not able to measure NfL values at 21 hpi in rats infected with serotype 8 due to high mortality in this group. Additionally, CSF tapping was difficult at 19 hpi likely due to the strong infection and swelling of the brain which was observed upon dissection. Values in this group may therefore be underestimates.

### Serotype 8 induced higher CSF concentrations of IL-6, IL-8 (GRO KC CINC-1), TNFα, IL-10, IL-1β in rats than did serotypes 15B and 14

**Figure 3:**
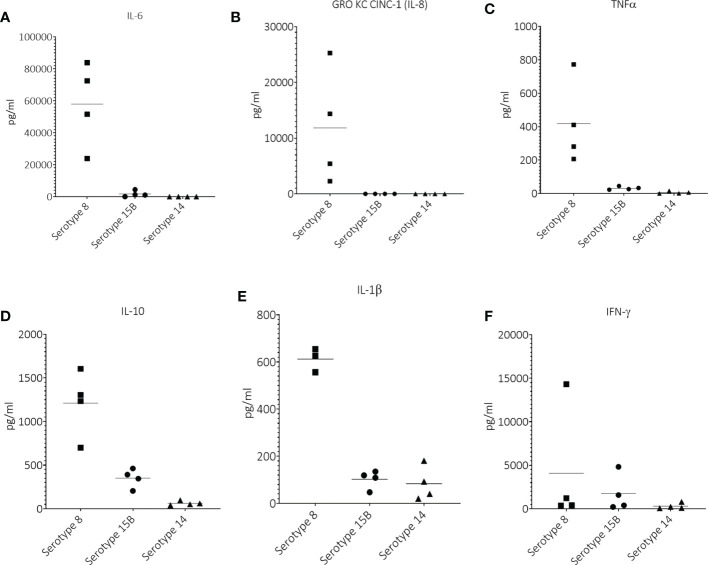
Cerebrospinal concentrations of **(A)** IL-6, **(B)** GRO KC CINC-1 (IL-8), **(C)** TNFα, **(D)** IL-10, **(E)** IL-1β and **(F)** IFN-γ at 19 hours post infection (hpi) in infant rats infected intracisternally with clinical pneumococcal strains of serotypes 8, 15B or 14. One point = one animal.

At 17 hpi, the only noticeable difference was a higher IFN-γ and IL-1β concentration in CSF of animals infected with serotype 8 and 15B compared to those infected with serotype 14 (**Supplementary Figure S1**). At 19 hpi, animals infected with serotype 8 had a notably higher average CSF concentration of IL-6, GRO KC CINC (IL-8), TNFα, IL-10 and IL-1β than those infected with serotype 15B or 14 (**Figures 3A–E**). The average CSF concentration of IFN-γ was only slightly higher for animals infected with serotype 8 or 15B compared to those infected with serotype 14 (**Figure 3F**). Cytokines at 21 hpi were not measured for serotype 8 infected animals due to high mortality in this group and difficulty obtaining CSF for analysis. At 21 hpi, animals infected with serotype 15B had higher concentrations of all measured cytokines compared to those infected with serotype 14 (**Supplementary Figure S2**). At 45 hpi and regardless of the infecting serotype, all tested cytokine values were below the limit of detection (data not shown).

### Fatal outcome for patients infected with strains of serotype 8 and 15B and raised inflammatory cytokine concentrations in all three patients

We looked at the clinical data for the three patients from which the isolates originated to see whether serotype 8 also caused severe disease in that patient and we observed the following. All patients were male and between 44 and 51 years of age. Both the patients infected with serotype 8 and with 15B had a fatal outcome. The patient infected with serotype 14 was discharged from hospital (**Supplementary Table S1**). All patients received antibiotic treatment. None had received a PCV vaccination prior to hospital admission. The patient infected with serotype 8 was living with HIV and tuberculosis positive. The patient infected with serotype 15B was also living with HIV and the patient with serotype 14 was neither HIV nor tuberculosis positive (**Supplementary Table 1**). All patients had inflammatory cytokine concentrations which were markedly higher than those reported in the literature as normal values in CSF (IL-6: 0 – 5 pg/ml; IL-8: 10 – 15 pg/ml; TNFα: 0 – 5 pg/ml; IL-10: 0 – 29 pg/ml; IFN-γ: 0 – 37 pg/ml; IL-1β: 0 – 4 pg/ml) (Kothur et al., 2016; Farrokhi et al., 2017; Liu et al., 2020) shown in **Supplementary Figure S3**. We did not perform statistical analysis on the values because there was one patient per serotype and because the patients may have been at different stages of disease.

### Possible mechanisms causing the hypervirulence of the serotype 8 isolate

Having concluded from the results above that the serotype 8 isolate was particularly virulent, next we aimed to uncover the possible mechanisms of its hypervirulence. As the capsule is a major virulence factor, we measured capsule size by FITC-dextran exclusion assay.

**Figure 4:**
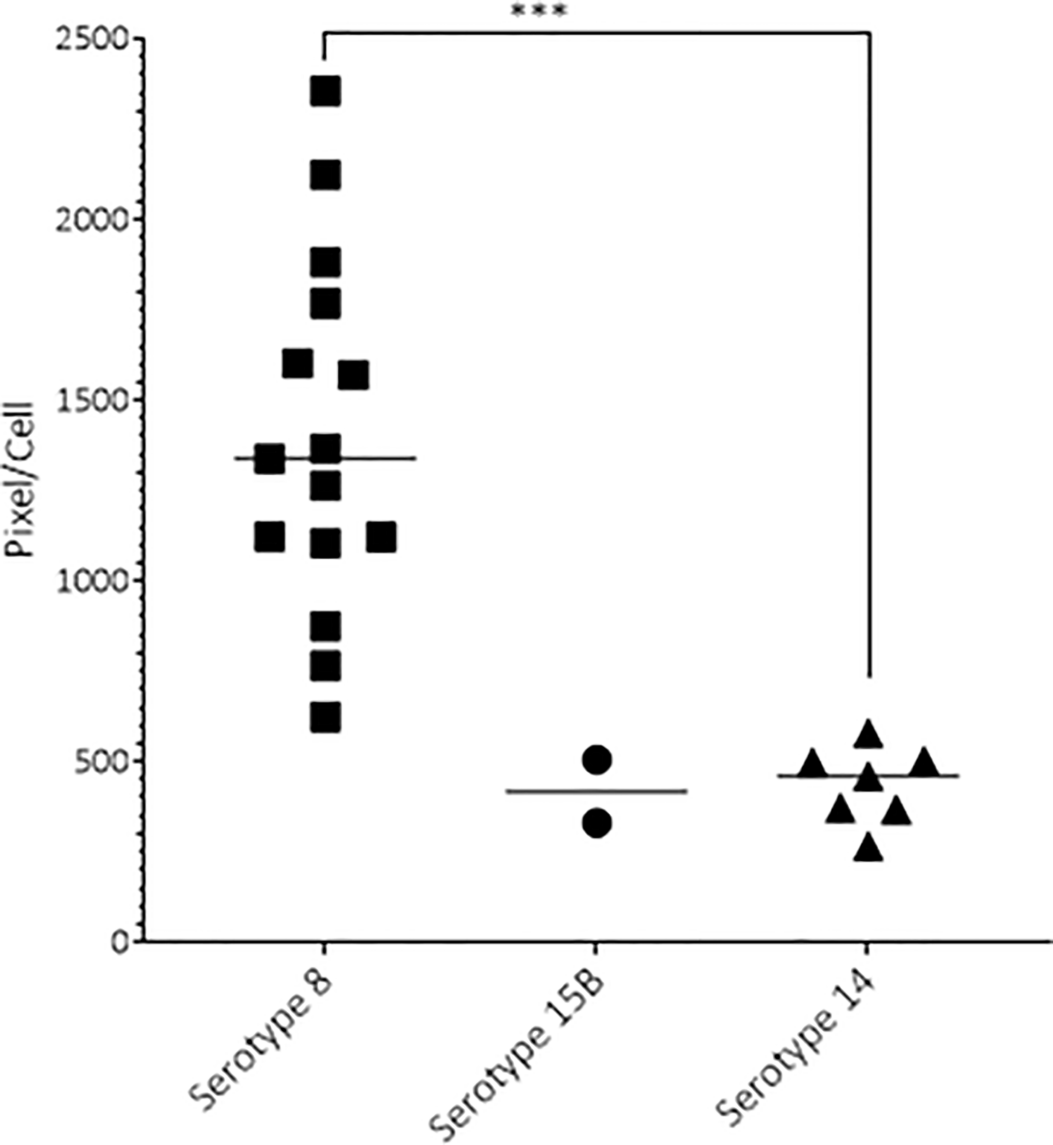
Capsule size of pneumococcal serotypes 8, 15B and 14 recovered 19 hours post infection. At 19 hpi, the serotype 8 strain had a significantly thicker capsule than the serotype 14 strain (p-value < 0.001). One point represents the average pixel/cell of one image with at least 1 bacterium. Animals infected with serotype 8 had more bacteria, thus more data points. ***p < 0.001.

Before inoculation of the rats, the average capsule size measured by FITC-dextran exclusion assay of serotype 8 (1311 pixel/cell, sd ± 283) was larger than that of serotype 15B (583 pixel/cell, sd ± 155, p<0.0001) or 14 (458 pixel/cell, ± sd 144, p<0.0001). We observed a similar pattern at 19 hpi where pneumococci recovered from the CSF of infected rats were larger for the serotype 8 isolate (1393 pixel/cell; sd ± 124) than that of the serotype 14 isolate (average 435 pixel/cell, sd ± 105, p < 0.001). Serotype 8 capsule was also thicker than that of serotype 15B, although not significantly due to the small number of bacteria recovered from the CSF (**Figure 4**). At 17 hpi we were not able to recover any bacteria from the group infected with serotype 14, most likely due to low bacterial concentration. At 17 hpi the average capsule size of serotype 8 (2220 pixel/cell, sd ± 1059) did not differ significantly to that of serotype 15B (1799 pixel/cell, sd ± 888). At 21 hpi we were unable to recover CSF for analysis of animals infected with serotype 8 due to high mortality and therefore capsule size analysis was not possible in this group. The capsule size of the serotype 15B and 14 strains did not differ significantly at 21 hpi. Capsule of serotype 8 and 15B were thicker, although not significantly, at 17 hpi compared to the inoculum. We did not observe a change in capsule size of serotype 14 at any of the time points. Analysis of the capsule size depended on the recovery of bacteria from CSF samples which proved to be challenging and was not successful for all infected animals. In summary, the serotype 8 isolate has a thicker capsule than the serotype 15B and 14 isolates.

**Figure 5:**
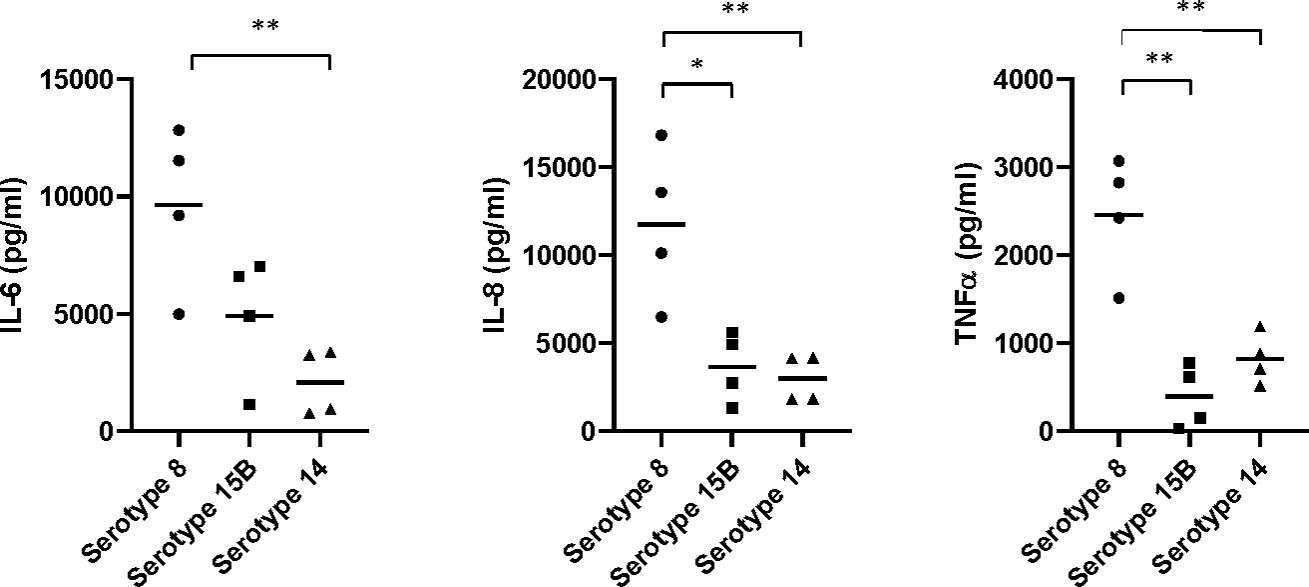
Neuroinflammatory response of mouse mixed glia to pneumococci of serotypes 8, 15B and 14. Release of pro-inflammatory cytokines (IL-6 and TNFα) and the neutrophil-attractant IL-8 (CXCL2) by glial cells (mixed cultures of astrocytes and microglia) in response to challenge with lysed bacteria. The values are from four independent experiments, the bar indicates the mean, * p<0.05, ** p<0.01.

Since inflammation is a major contributor to severity of pneumococcal meningitis, we assessed whether the inflammatory cytokine response of mouse brain glia cells differed in response to the three isolates.The mean concentration of IL-6 released by primary mouse glia cells was higher in response to lysate of the serotype 8 isolate than to either the serotype 15B isolate (not significant) or serotype 14 isolate (p<0.01). Similarly, the concentration of IL-8 was significantly greater in response to the serotype 8 isolate than the serotype 15B (p<0.05) or 14 (p<0.01) isolates. The same was true for TNFα: serotype 8 *vs* serotype 15B (p<0.01); serotype 8 *vs* 14 (p<0.01) (**Figure 5**). Therefore, primary mouse-glia release more IL-6, IL-8 and TNFα in response to the serotype 8 pneumococcal isolate than the serotype 15B or 14 isolates.

**Figure 6:**
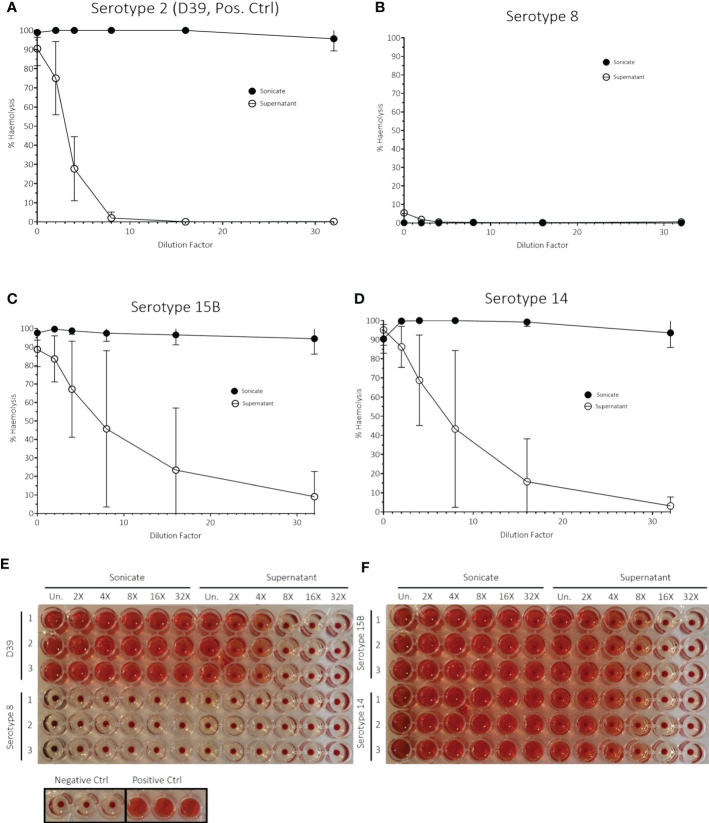
Haemolytic activity assay with human blood of **(A)** D39, serotype 2 (positive bacteria control) and strains used in *in vivo* experiments of **(B)** serotype 8, **(C)** serotype 15B and **(D)** serotype 14. The data represent three independent experiments (n = 3 replicates, mean ± sd). The assay was carried out with both sonicated bacteria (= sonicate) and the supernatant of bacterial cultures in brain-heart infusion broth (BHI) (= supernatant). dH_2_O water was used as a positive control. Phosphate buffered saline (PBS, pH = 7.0) was used as a negative control. Images **(E, F)** show results of the haemolysis experiment after centrifugation. All experiments were performed in triplicates (1, 2, 3). rows: from left to right: dilution from undiluted sample (Un.) is indicated.

**Figure 7:**
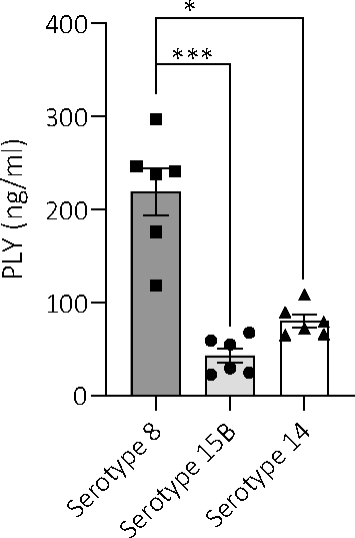
Amount of pneumolysin in total lysates of serotypes 8, 15B and 14. ELISA-based quantification of the amount of pneumolysin released by the three different serotypes (at a bacterial concentration of 5 x 10^6^ CFU/ml) upon lysis by penicillin treatment reveals significantly higher levels of the toxin in serotype 8, compared to serotype 15B and 14. Data are represented as mean values. Samples were quantified in triplicates from two independent preparations, *p < 0.0004, ***p < 0.0001.

Another factor associated with virulence is haemolysis caused by pneumolysin toxin and so we measured the haemolytic activity of the three isolates. The serotype 8 sonicate showed no haemolytic activity when incubated with human erythrocytes and the supernatant had only 5% haemolytic activity when undiluted. This haemolytic activity disappeared at a 6X dilution. In contrast, the serotype 15B and 14 isolates, as well as the D39 serotype 2 (positive control), all showed 100% haemolysis of the sonicated bacteria pellet, even at the highest dilution tested (**Figure 6**). Although there were large standard deviations for some dilutions for the serotype 15B and 14 isolates, which could be due to difference in bacterial numbers between the experiments, it was nevertheless clearly evident that the serotype 8 isolate differed from the other strains regarding haemolytic activity. As the hypervirulent serotype 8 isolate is non-haemolytic in human blood, we measured whether it produces pneumolysin and found that the serotype 8 isolate released more pneumolysin upon lysis by penicillin/streptomycin treatment than the serotype 15B and 14 isolates (**Figure 7**).

**Figure 8:**
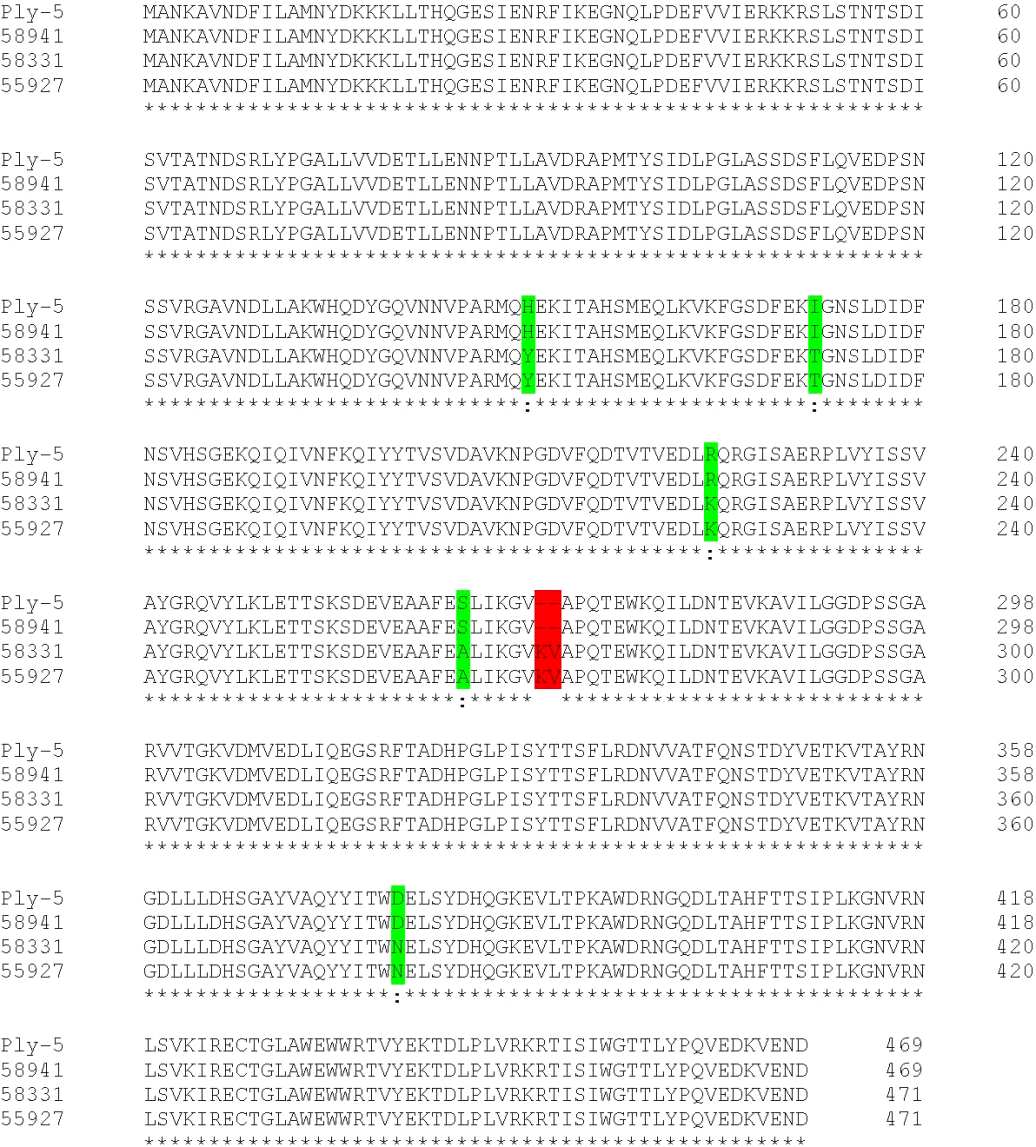
Amino acid sequence alignment of ply-5 reference sequence, serotypes 8 (58941), 15B (58331) and 14 (55927) *ply* alleles. Substitutions in the serotype 8 isolate relative to the other two clinical isolates are indicated in green and a deletion is indicated in red.

Since the hypervirulent serotype 8 isolate showed no haemolytic activity in human blood but did produce pneumolysin, we determined its pneumolysin allele. We sequenced the genome and compared the pneumolysin allele to that of the *S. pneumoniae* strain 01-1199 which has the pneumolysin allele ply-5 and has been described to be non-haemolytic (Jefferies et al., 2007). We found that the amino acid sequence of the ply allele of the serotype 8 tested in our study had a 100% sequence identity to that of ply-5 allele, encoding pneumolysin which has lost its pore-forming activity (Badgujar et al., 2020) (**Figure 8**) and differs from that of serotypes 15B and 14 by amino acid substitutions at positions 150 (Y to H), 172 (T to I), 224 (K to R), 265 (A to S) and a deletion of KV at position 271 to 272.

### Liposomes attenuated the IL-6, TNFα and IL-8 response from mixed glia cells

**Figure 9:**
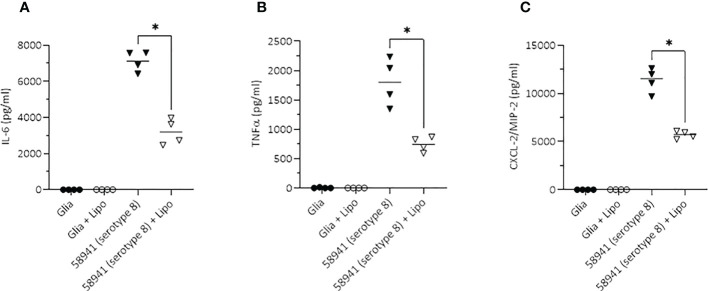
Inhibitory effect of liposomes on the neuroinflammatory response of mouse glial cells. Scavenging of the non-haemolytic pneumolysin of the serotype 8 strain 58941 by liposomes significantly reduced the pro-inflammatory response (determined by induction of the two major pro-inflammatory cytokines IL-6 and TNFα, and the chemokine CXCL-2/MIP2 corresponding to the human IL-8) in an *in vitro* meningitis model. Lytic release of PLY was achieved by treatment of the bacteria with penicillin during incubation with the glial cells. All values represent the mean, n = 4 independent experiments, *p<0.05.

To test whether liposomes, designed to sequester pneumolysin, reduce the neuroinflammatory response to the non-haemolytic pneumolysin-producing hypervirulent serotype 8, we incubated primary mouse glia cells with the pneumococci in the absence and presence of liposomes. The liposomes significantly reduced the IL-6, TNFα and CXCL2/MIP-2 (corresponding to human IL-8) responses (p < 0.05) (**Figure 9**).

## Discussion

In this study, we analysed CSF samples from three pneumococcal meningitis patients from South Africa each infected with one of three different serotypes of high epidemiological importance and tested the pathologic profile of the isolates in *in vitro* and *in vivo* models. One of the three isolates was unexpectedly virulent in the infant rat model of meningitis causing more severe and rapid disease than we have previously observed with any unpassaged clinical pneumococcal isolate. This isolate was of serotype 8, ST 53, isolated from a patient with fatal disease outcome.

We found that this serotype 8 isolate produced a non-haemolytic form of pneumolysin and had a thicker capsule compared to the serotype 15B and 14 isolates tested. Both virulence factors are known to play essential roles in disease manifestation and pneumolysin, in particular, contributes to the neuroinflammatory response and severe disease outcome (Wellmer et al., 2002; Hirst et al., 2008; Hupp et al., 2022). In the rat model, the severity of disease caused by the serotype 8 isolate correlated with its induction of the highest concentrations of inflammatory cytokines of the isolates tested. This result was also supported by the *in vitro* model of primary cells isolated from the brains of mice where the secretion of IL-6, IL-8 and TNFα was greater from a mixed culture of astrocytes and microglia exposed to lysates of serotype 8 than to the serotype 15B or 14 isolates. The thicker polysaccharide capsule protects the bacteria from the host immune response and phagocytosis by professional phagocytes such as microglia (brain resident macrophages), due to impaired opsonization and subsequent inhibition of complement activation (Hyams et al., 2010). We propose that this will promote bacterial proliferation and in turn result in release of high amounts of bacterial lytic factors contributing to an enhanced proinflammatory response.

We could not see the same pattern of cytokines in the CSF from the patients but the concentrations found in the human CSF samples resemble that of a cytokine storm. The cytokine profiles in the CSF of the meningitis patients may no longer reflect the early acute phase of disease as patients may be admitted to hospital at a progressed, severe stage of disease.

The patients infected with the serotype 8 and 15B isolates had a fatal outcome whereas the patient infected with the serotype 14 isolate was discharged from hospital. A limitation of this data is that it is only derived from one patient for each serotype and that only the serotype 14 came from a HIV negative patient. However, our previous large epidemiology study (Müller et al., 2022) showed that serotype 8 pneumococci are the most commonly found serotype in IPD patients in South Africa in the vaccine era and that serogroup 15 was associated with increased case-fatality ratio (CFR) whereas serotype 14 was less associated with CFR (Müller et al., 2021). In the rat model, both the serotype 8 and, to a lesser extent, 15B isolate caused more severe disease than the serotype 14, in the absence of the confounding factors of HIV infection, diverse host genetic background and stage of infection. We determined disease severity by mortality, apoptosis in the hippocampus and volume of cortical damage. However, we did not see a higher concentration of neurofilament light chain (NfL) in the CSF of rats infected with the serotype 8 strain. A previous study concluded that measuring NfL is a very sensitive method to detect neuronal damage (Le et al., 2020). The apparent lack of NfL induced by the serotype 8 strain may potentially be due to a selection bias. We do not have data for the later timepoints when NfL would be expected to have accumulated in the group infected with serotype 8 because of the high mortality at the early stages of infection for this group.

It has been reported that capsule size could be an indicator for disease severity (Hathaway et al., 2016; Muller et al., 2020) and this fits in with our observation in this study that the serotype causing the most mortality in the meningitis *in vivo* model is also the one with the thickest capsule before and during infection. We suspected that not only the capsule but also a strong haemolytic activity of the pneumolysin of this strain could contribute to the disease severity. However, we found the serotype 8 to have no detectable haemolytic activity. Sequencing of the pneumolysin gene confirmed that this strain harboured a non-haemolytic pneumolysin. Non-haemolytic pneumolysin has been found to be associated with meningitis outbreaks in serotype 1 and has already been reported in recent publications in serotype 8 strains (Kirkham et al., 2006; Jefferies et al., 2007; Panagiotou et al., 2020). Although the serotype 8, ST53, strain had no haemolytic activity, we found it produced large quantities of pneumolysin, as seen with serotype 1 strains with increased pathogenicity (Jacques et al., 2020). Additionally, a recent publication showed that pneumolysin with a mutation at position 150 led to a loss of the pore-forming activity (Badgujar et al., 2020) which improved cellular invasion and autophagy evasion and therefore promoted an intracellular lifestyle. As the allele in our tested strain also has this point mutation, we suspect that this serotype 8 strain has also lost the pore-forming activity which needs to be confirmed. The loss of lytic activity of pneumolysin in combination with the thick serotype 8 capsule could render this strain particularly virulent once it invades and causes IPD.

Liposomes, rather than reducing the number of bacteria, act by sequestering pneumolysin and thus reduce the inflammatory response towards haemolytic pneumolysin. However, we were unaware of any studies on their effect on non-haemolytic pneumolysin. We therefore tested the effect of liposomes on the ability to reduce the cytokine response in an *in vitro* co-culture model with murine glia cells. Addition of liposomes reduced the cytokine concentrations in response to the serotype 8 strain indicating that liposomes are still able to sequester this non-haemolytic form of pneumolysin.

Our study combined patient data with experimental *in vivo* and *in vitro* data with an emphasis on the experimental data due to the limited number of patients. Our experimental model was based on the injection of bacteria directly into the CSF which does not reflect the natural course of infection. However, rodent meningitis models are well accepted and widely used experimental setups to investigate bacterial meningitis. For the human data, there was only one patient per serotype and we were not able to determine and control the time point at which the patients were admitted to the hospital and which could have an important effect on the CSF cytokine profile and concentration. Comparison between patients is also complicated by the individuals’ different genetic backgrounds and additional severe health conditions particularly HIV and tuberculosis. Therefore, support of clinical data with experimental data is of high importance.

We note that a recent publication has described serotype 8 as an invasive serotype in both children and adults and that invasiveness is primarily determined by serotype but is modified by strain (Lochen et al., 2022). In this context, the combination of our data from the *in vivo* rat model and *in vitro* primary mouse brain cells, as well as patient data, leads us to propose that it is worth paying particular attention to serotype 8 strains of ST 53, possessing a thick capsule and non-haemolytic pneumolysin. Serotype 8 is not included in conjugate vaccines in current use and is the most common serotype causing IPD in several populations (Sanz et al., 2019; Oyewole et al., 2021; Hansen et al., 2021; Müller et al., 2022) and the strain used here appeared to be hypervirulent. It is therefore encouraging to consider that liposome-based therapy, designed to sequester haemolytic pneumolysin, also appears to have therapeutic potential for non-haemolytic, but highly virulent, pneumococcal strains.

## Data availability statement

The original contributions presented in the study are publicly available. This data can be found here: https://www.ncbi.nlm.nih.gov/search/all/?term=PRJNA912134.

## Ethics statement

All protocols of the GERMS-SA surveillance study were approved by the Human Research Ethics Committee (Medical), University of Witwatersrand (clearance numbers M140159, M081117, M021042). Additional ethical permission for the collection of CSF samples was granted by the Human Research Ethics Committee (Medical), University of Witwatersrand (clearance number M180101). Where required, approval was granted by provincial and university ethics committees (Clearance numbers: Free State 230408-011/180/09A; Kwazulu-Natal BF130/11 and HRKM024/09; Western Cape REC REF 115/2009, N04/01/001). Permission for patient data analysis in Switzerland was obtained from the Cantonal Ethics Commission Bern (Kantonale Ethikkommission für die Forschung, KEK, Bern, project number 2018-01172). Informed consent for analysis of CSF was collected from patients and/or guardians for both collection of data and analysis of CSF. All research was performed in accordance with the relevant guidelines and regulations. The patients/participants provided their written informed consent to participate in this study. The animal study was reviewed and approved by the Animal Care and Experimentation Committee of the Canton of Bern Switzerland (license no. BE91/20 and BE76/14) and followed the Swiss national guidelines for the performance of animal experiments.

## Author contributions

LJH and AG conceived, designed and coordinated the study. AM performed haemolysis experiments. AM analysed, interpreted data and made tables and figures for *in vivo* and human data. CL performed WGS analysis. AM, CL and MP interpreted WGS results. SH measured Ply concentrations, performed *in vitro* experiments with primary mouse glial cells and made the corresponding figure, supported by AI. EB provided liposomes and LH performed ELISAs. The draft manuscript was written by AM. AM and LJH designed experiments. DG and SL helped design and supported *in vivo* experiments. All authors contributed to the article and approved the submitted version.
