# Supplementary material for: Meningitis-associated pneumococcal serotype 8, ST 53, strain is hypervirulent in a rat model and has non-haemolytic pneumolysin which can be attenuated by liposomes
Source: Front Cell Infect Microbiol. 2023 Jan 6;12:1106063. doi: 10.3389/fcimb.2022.1106063 (PMC9852819; doi:10.3389/fcimb.2022.1106063)
Supplement: Supplementary file 1 [file DataSheet_1.pdf]

Supplementary Table S1 Basic clinical data of patients infected with strains used for lab experiments

	Serotype 8	*Serotype 15B*	*Serotype 14*
In-hospital outcome	Died	Died	Discharged
Age	51	44	49
Gender	Male	Male	Male
HIV status	positive (WHO stage 4)	positive (WHO stage 4)	negative
HIV markers	candida, wasting, necrotic skin	candida, wasting	Not applicable
If HIV, CD4 count (cell/mm^3)	5	Unknown	Not applicable
Antiretroviral therapy	No	Unknown	Not applicable
Tuberculosis	positive	No	No
Diagnosis	Meningitis and LRTI	Meningitis	Meningitis
GCS	3*	13	14
PCV vaccination received	no	no	no
CSF neutrophil (cells/µl)	30	42	118
CSF lymphocytes (cells/µl)	88	0	12
CSF erythrocytes (cells/µl	0	28	19
CSF proteins (g/dL)	1.34	4.64	1.37
CSF glucose (mmol/L)	0.3	0.001	0.1
Antibiotic treatment	Yes	Yes	Yes

HIV: human immunodeficieny virus; WHO: World Health Organization; LRTI: Lower respiratory tract infection; GCS: Glasgow coma scale; PCV: polysaccharide conjugate vaccine; CSF: cerebrospinal fluid

*GCS was reported as 2. Reported as 3 here as 3 is the minimum.

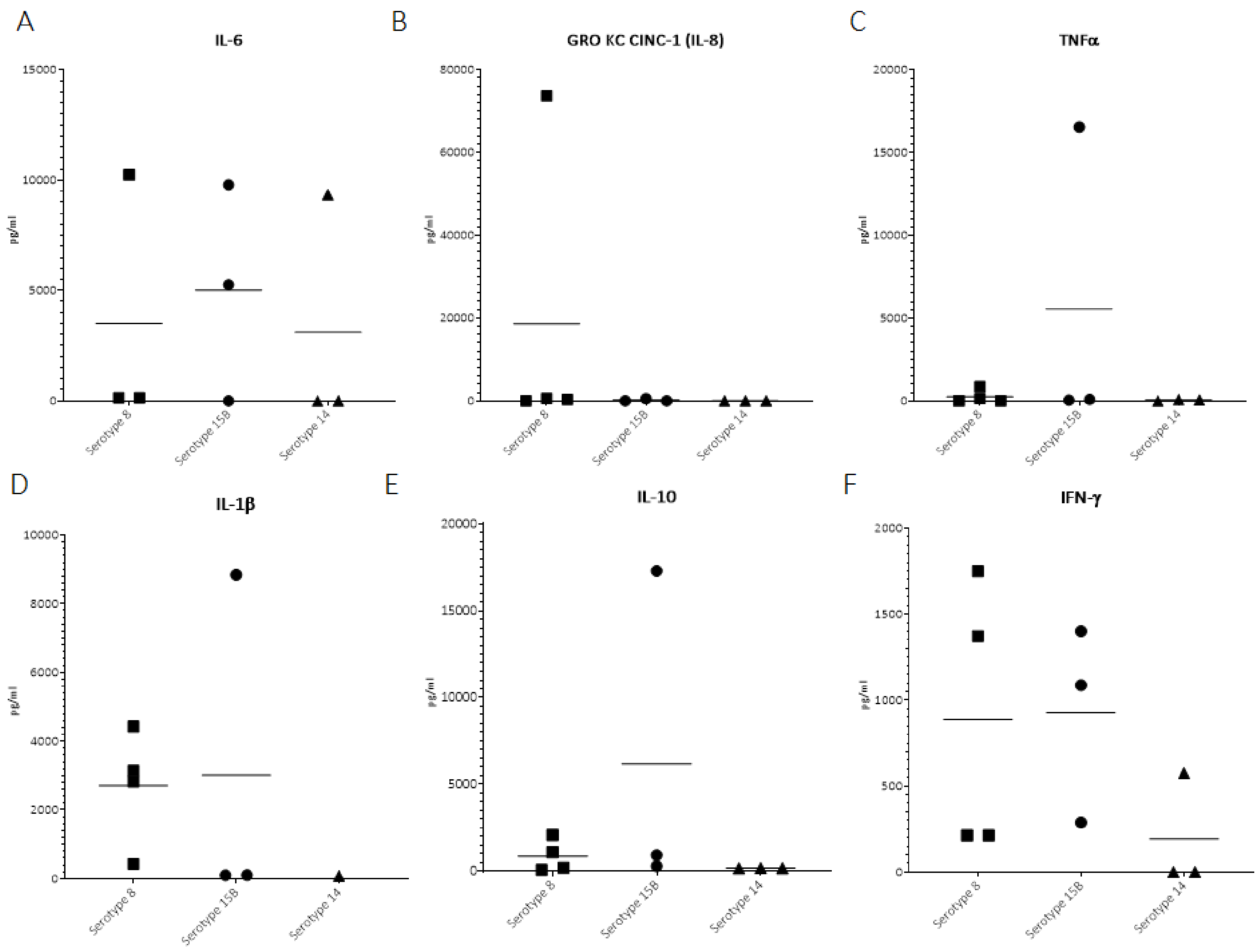

Supplementary Figure S1 Cerebrospinal concentrations of (A) IL-6, (B) GRO KC CINC-1 (IL-8), (C) TNFα, (D) IL-10, (E) IL-1β and IFN-γ at 17 hours post infection (hpi) in infant rats infected intracisternal with clinical pneumococcal strains of serotypes 8, 15B or 14. One point = one animal

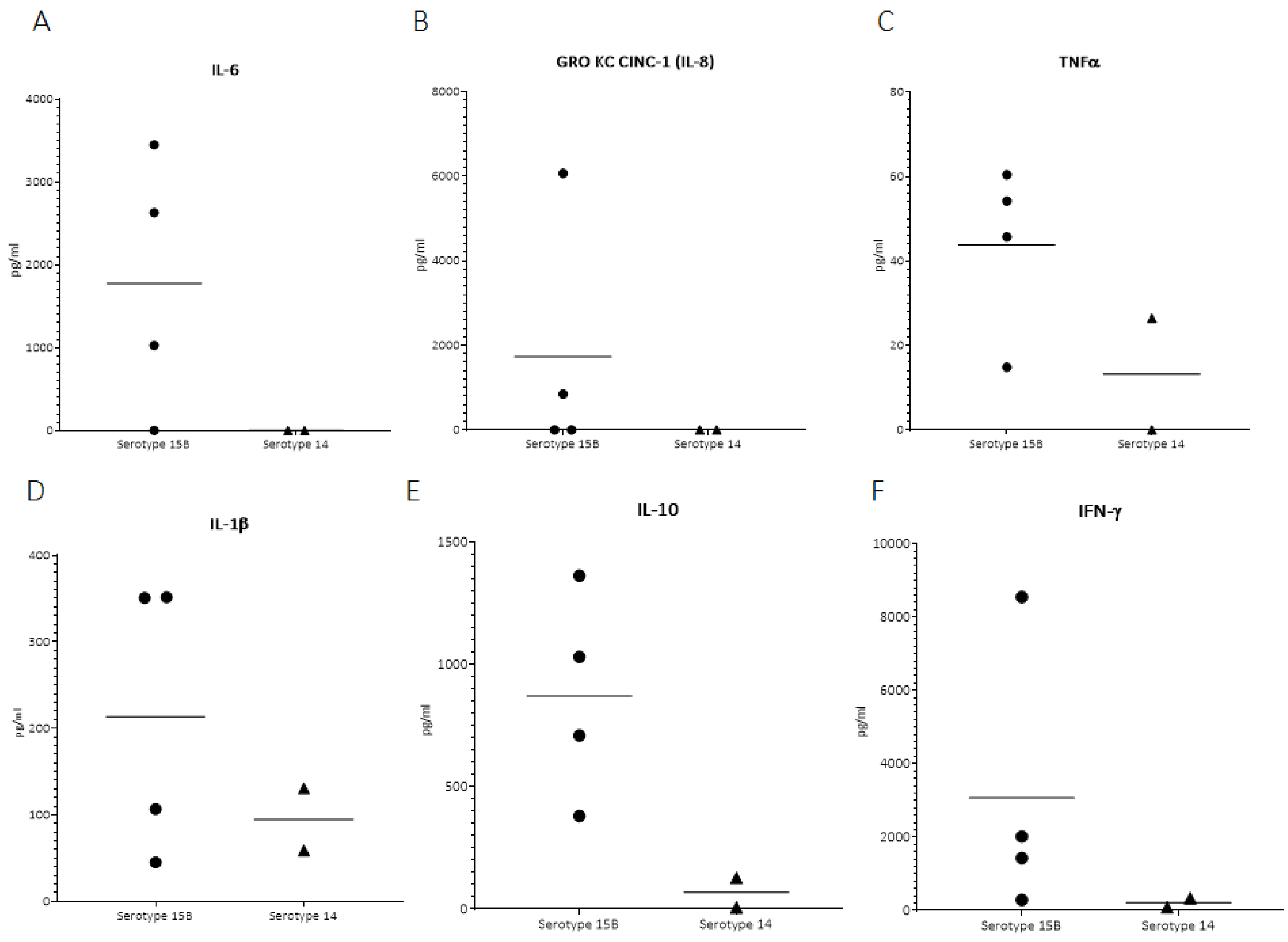

Supplementary Figure S2 Cerebrospinal concentrations of (A) IL-6, (B) GRO KC CINC-1 (IL-8), (C) TNFα, (D) IL-10, (E) IL-1β and IFN-γ at 21 hours post infection (hpi) in infant rats infected intracisternal with clinical pneumococcal strains of 15B or 14. Animals infected with serotype 8 are not shown due to spontaneous death of all animals before end of experiment and unavailability of CSF to perform cytokine analysis. One point = one animal

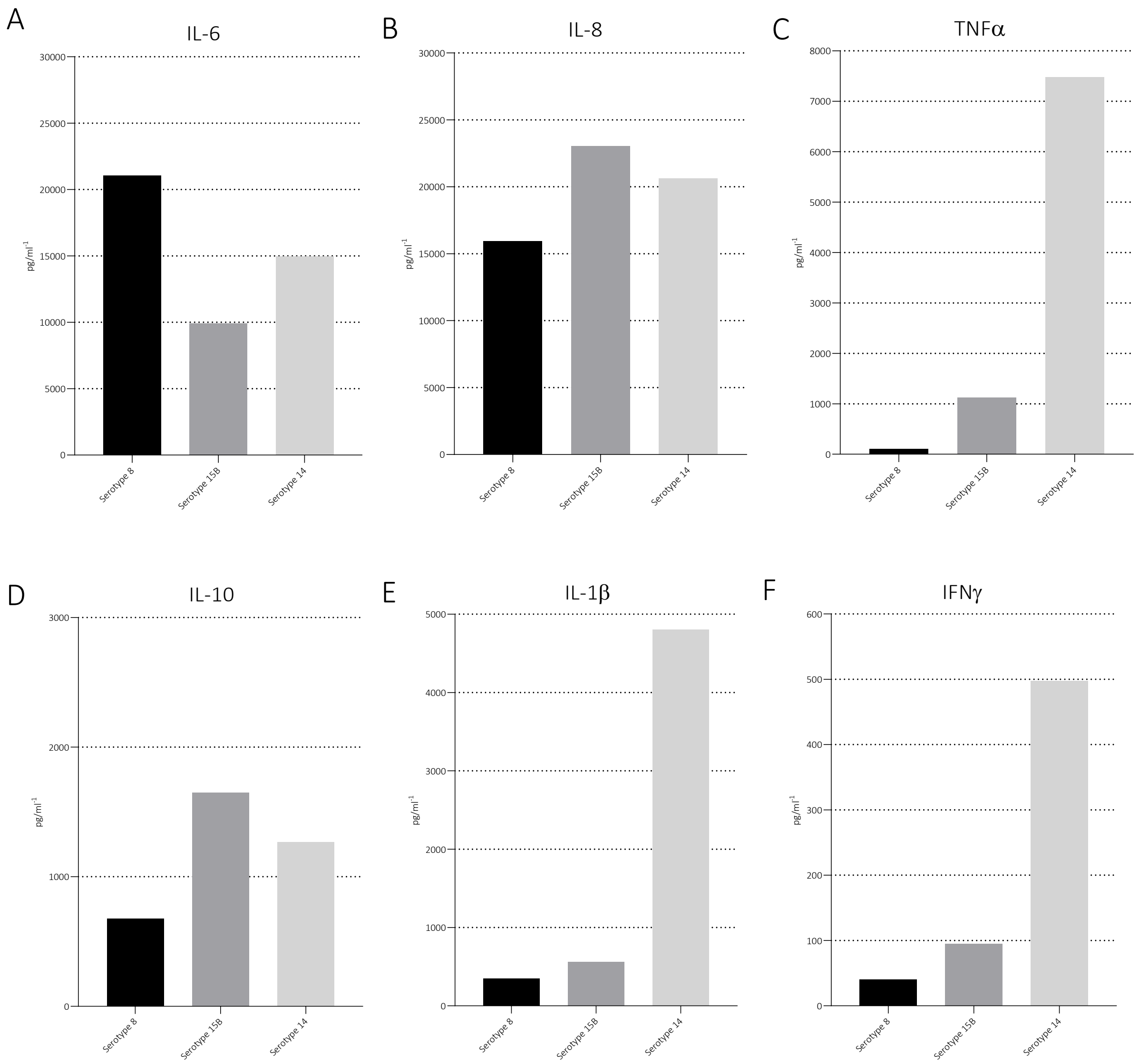

Supplementary Figure S3 Cerebrospinal fluid concentrations of (A) IL-6, (B) IL-8, (C) TNFα, (D) IL-10, (E) IL-1β and (F) IFNγ in patients with confirmed meningitis of pneumococcal serotypes 8, 15B or 14. One bar represents one patient.
